# Hospital and laboratory outcomes of patients with COVID-19 who received vitamin D supplementation: a systematic review and meta-analysis of randomized controlled trials

**DOI:** 10.1007/s00210-022-02360-x

**Published:** 2022-12-12

**Authors:** Mohamed Sayed Zaazouee, Mahmoud Eleisawy, Amira M. Abdalalaziz, Mahmoud M. Elhady, Omar Adel Ali, Taghreed Mohamed Abdelbari, Sara Mohamed Hasan, Hossam Waleed Almadhoon, Alaa Yehia Ahmed, Alaa Shaban Fassad, Rewan Elgendy, Eman Adnan Abdel-Baset, Hamis A. Elsayed, Ahmed Bostamy Elsnhory, Alaa Bahaaeldin Abdraboh, Hazem Metwally Faragalla, Alaa Ahmed Elshanbary, Osama A. Kensara, Mohamed M. Abdel-Daim

**Affiliations:** 1 Faculty of Medicine, Al‐Azhar University, Assiut, Egypt; 2 Faculty of Medicine, Benha University, Benha, Egypt; 3 Faculty of Medicine, Alexandria University, Alexandria, Egypt; 4 Faculty of Medicine, Ain Shams University, Cairo, Egypt; 5 Institute of Biodiversity, One Health and Veterinary Medicine, University of Glasgow, Glasgow, UK; 6 Faculty of Medicine, Mansoura University, Mansoura, Egypt; 7 Faculty of Medicine, Al-Azhar University, Cairo, Egypt; 8 Faculty of Medicine, Modern University for Technology and Information (MTI), Cairo, Egypt; 9 Department of Clinical Nutrition, Faculty of Applied Medical Sciences, Umm Al-Qura University, Makkah, Saudi Arabia; 10 Department of Pharmaceutical Sciences, Pharmacy Program, Batterjee Medical College, Jeddah, Saudi Arabia; 11 Pharmacology Department, Faculty of Veterinary Medicine, Suez Canal University, Ismailia, Egypt

**Keywords:** COVID-19, Vitamin D, Hospital outcomes, Laboratory outcomes, Meta-analysis

## Abstract

Coronavirus disease 2019 (COVID-19) has a wide-ranging spectrum of clinical symptoms, from asymptomatic/mild to severe. Recent research indicates that, among several factors, a low vitamin D level is a modifiable risk factor for COVID-19 patients. This study aims to evaluate the effect of vitamin D on hospital and laboratory outcomes of patients with COVID-19.

Five databases (PubMed, Embase, Scopus, Web of Science, and Cochrane Library) and clinicaltrials.gov were searched until July 2022, using relevant keywords/Mesh terms. Only randomized clinical trials (RCTs) that addressed the topic were included. The Cochrane tool was used to assess the studies’ risk of bias, and the data were analyzed using the review manager (RevMan 5.4).

We included nine RCTs with 1586 confirmed COVID-19 patients. Vitamin D group showed a significant reduction of intensive care unit (ICU) admission (risk ratio = 0.59, 95% confidence interval (CI) [0.41, 0.84], *P* = 0.003), and higher change in vitamin D level (standardized mean difference = 2.27, 95% CI [2.08, 2.47], *P* < 0.00001) compared to the control group. Other studied hospital and laboratory outcomes showed non-significant difference between vitamin D and the control group (*P* ≥ 0.05).

In conclusion, vitamin D reduced the risk of ICU admission and showed superiority in changing vitamin D level compared to the control group. However, other outcomes showed no difference between the two groups. More RCTs are needed to confirm these results.

**Supplementary Information:**

The online version contains supplementary material available at 10.1007/s00210-022-02360-x.

## Introduction

Coronavirus disease 2019 (COVID-19) is generated by the novel beta coronavirus known as severe acute respiratory syndrome coronavirus 2 (SARSCoV2). The disease already had spread across the globe and leading the World Health Organization to declare it a pandemic. Since then, more than 508 million proven cases and 6 million mortalities have been reported through April 26, 2022 (Dong et al. 2020; Hu et al. 2021a; Cannata-Andía et al. 2022). COVID-19 has a wide spectrum of clinical symptoms, from asymptomatic or milder symptoms with fever, tiredness, and dry cough to severe and critical symptoms with dyspnea, intensive care unit (ICU) admission, acute respiratory distress syndrome, and multiorgan damage. Immunodeficiency, black ethnicity, older age, chronic kidney disease, obesity, and chronic metabolic disorders are risks related to COVID-19 severity (Xie et al. 2020; Hu et al. 2021b; Olumade and Uzairue 2021; Zhang et al. 2021; Guan et al. 2020).

Vitamin D deficiency was linked to the severity of viral diseases like influenza (Watkins et al. 2015). Recent research indicates that, among several factors, a low vitamin D level is a risk factor that can be modified for COVID-19 patients (Borna et al. 2022; Ilie et al. 2020; Grant et al. 2020; Meltzer et al. 2020). Vitamin D is known to have an antiinflammatory effect, modulate innate and adaptive immunological responses, and enhance the volume of antimicrobial proteins (AlSafar et al. 2021; Pinheiro et al. 2021; Malek Mahdavi 2020; Gois et al. 2017). According to new evidence, it possibly mitigates SARS-CoV-2 expression of the gene and reduces infection by binding to its receptor (Brito et al. 2021b; Glinsky 2020). However, there is no conclusive proof of vitamin D’s preventative and therapeutic significance in COVID-19 (Brito et al. 2021a).

Despite vaccine releases, considerable attention has been devoted to further preventive strategies, like vitamin D supplementation. Some studies showed the effectiveness of vitamin D in COVID-19, and they recommended it as a possible way of improving immune responses to COVID-19 vaccination (AlSafar et al. 2021; Graham 2020; Pinheiro et al. 2021; Malek Mahdavi 2020; Velikova et al. 2021). Also, some observational studies linked a lower vitamin D level to COVID-19 predisposition, morbidity, and mortality consequences (Angelidi et al. 2021; Bychinin et al. 2021; Campi et al. 2021; Infante et al. 2022; Orchard et al. 2021). However, there is no definite evidence of vitamin D supplementation’s beneficial and protective use in COVID-19 (Mercola et al. 2020; Petrelli et al. 2021; Fernandes et al. 2022; Rastogi et al. 2020; Tentolouris et al. 2022; Varikasuvu et al. 2022b; Cannata-Andía et al. 2022; Murai et al. 2021a; Soliman et al. 2021; Elamir et al. 2022; Cui et al. 2022). Therefore, this study aims to assess the effect of vitamin D on hospital and laboratory outcomes of COVID-19 patients.

## Materials and methods

We depended on the PRISMA-P statement and the guideline of the Cochrane handbook for systematic reviews during this systematic review and meta-analysis (Higgins et al. 2019; Page et al. 2021).

### Searching databases and keywords

Clinicaltrials.gov registry and five databases (Embase, Web of Science, PubMed, Scopus, and Cochrane Library) were searched until July 2022. We used the following search terms: “COVID 19,” “SARS CoV 2 Infection,” “COVID-19,” “Coronavirus,” “SARS-CoV-2 Infection,” “2019-nCoV Disease,” “SARS,” “Severe Acute Respiratory Syndrome,” “COVID19,” “Vitamin D,” “Calciol,” “Vitamin D 3,” “Vitamin D3,” “Cholecalciferol,” “25 Hydroxyvitamin D3,” “Calcidiol,” “25 Hydroxycholecalciferol,” “Calcifediol,” “Dedrogyl,” “Hydropherol,” “Calderol”. The search was not limited to any time or language. The above electronic search was complemented with a manual search in the reference records of included studies.

### Eligibility criteria and study selection

All RCTs (S) that reported on COVID-19 patients (P) who received vitamin D supplementation (any type) (I) and compared their hospital and laboratory outcomes (O) with similar patients who received no intervention/placebo (C). Two types of outcomes were of this review focus as the following:

- Primary outcomes (hospital): The need for ICU admission, ventilation and oxygen therapy, the risk of death, and the length of hospital stay (days).
- Secondary outcomes (laboratory): The level of C-reactive protein (mg/dL), interleukin-6 (pg/mL), vitamin D concentration, lactate dehydrogenase (LDH), calcium concentration, creatinine, d-dimer, neutrophil count, lymphocyte count, platelet count, and leucocytes (no./μL).

Studies of other designs were excluded, including case reports, case series, reviews, editorials, in vitro, postmortem, conference abstract, letters to the editor, and author opinion papers. Titles and abstracts of potentially included studies were screened to include relevant ones, and then the full-texts were reviewed thoroughly to confirm the eligibility to be finally included. Four independent authors conducted the previous two steps, but in cases of indecision, a supervisor was involved to confirm the decision.

### Data extraction and risk of bias assessment

Three authors extracted the following baseline items from the included trials: (a) general data: study arms, sample size, sex, age, and body mass index (BMI) (kg/m^2^) of participants; (b) comorbidities outcomes: diabetes, chronic obstructive pulmonary disease, hypertension, cardiovascular disease, and asthma; and (c) common COVID-19 symptoms: fever, cough, weakness, and diarrhea. Another three reviewers extracted the following summary data from the included trials, including NCT, vitamin D administration, follow-up period, and study’s primary outcomes and main findings. Six authors extracted the outcomes mentioned above.

The quality of the RCTs was appraised independently by five co-authors using the Cochrane tool to assess the risk of bias reported in the Cochrane Handbook for Systematic Reviews (part 2, chapter 8.5), which categorized the evaluated studies into three categories: high, low, or unclear risk. Indecisions, if any, were resolved through discussion and consensus with six co-authors.

### Statistical analysis

We conducted this meta-analysis using Review Manager Software 5.4. Continuous outcomes were pooled as mean difference (MD) and 95% confidence intervals (CIs). In case of different assessment tools/devices, the data were pooled as standardized mean difference (SMD). Dichotomous outcomes were pooled as risk ratio (RR) and 95% CI. We pooled the data under the fixed-effect model and tested the heterogeneity between pooled studies by *X*^2^ and *I*^2^ tests. Once the heterogeneity between studies was detected (*P*-value < 0.1 and *I*^2^ > 50%), a random-effect model was used. We tried to solve the heterogeneity by sensitivity analysis using the leave-one-out method. The data were considered statistically significant if *P*-value < 0.05. Since the number of the included studies (*n* = 9) is less than 10, the publication bias could not be evaluated, according to Egger et al. (1997).

## Results

### Literature search

**Fig. 1:**
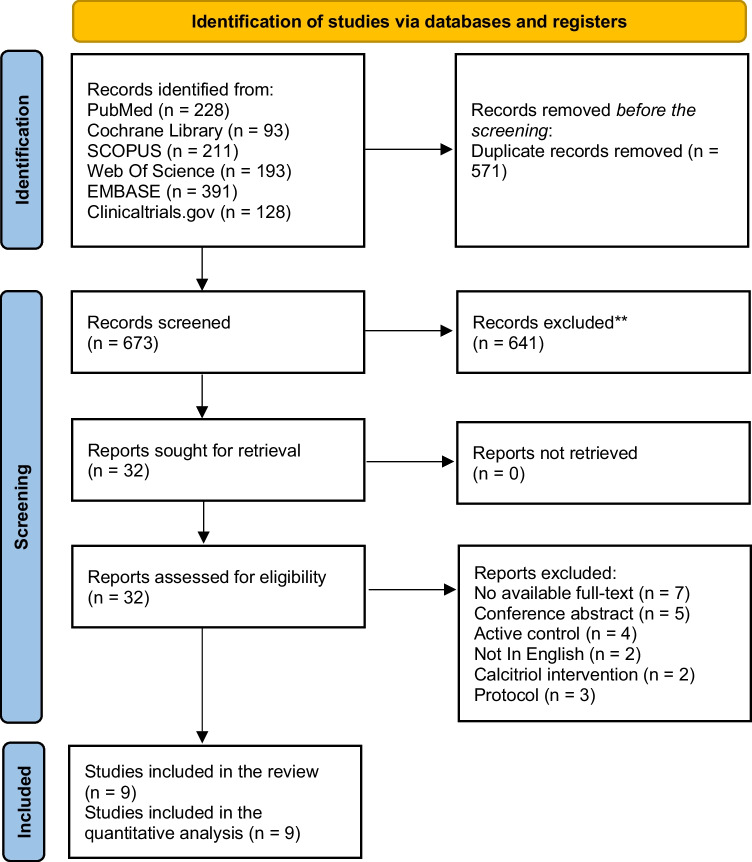
The PRISMA flow diagram of the included studies

We retrieved 1244 records through an extended literature search on different search engines and excluded 571 papers by duplicate removal. The title and abstract screening excluded 641 articles. Thirty-two articles underwent full-text screening, and nine RCTs matched our criteria and entered all steps of meta-analysis to get the evidence (Cannata-Andía et al. 2022; Entrenas Castillo et al. 2020; Fernandes et al. 2022; Maghbooli et al. 2021; Murai et al. 2021b; Rastogi et al. 2022; Mariani et al. 2022; Karonova et al. 2022; Soliman et al. 2021). See the PRISMA chart in Fig. 1.

### Characteristics of included studies

We included all RCTs that studied the effect of vitamin D on hospital and laboratory outcomes of 1586 confirmed COVID-19 patients with a mean (SD) age of 56.41 (11.69). The intervention and control groups sample ranged from 16 to 274 participants, and males were more prominent than females. COVID-19 symptoms varied among patients, including fever, cough, general weakness, and diarrhea. Most patients received oral administration regarding vitamin D supplementation, but a single group received an intramuscular injection. Most participants suffered from comorbidities such as hypertension, diabetes, or asthma. Researchers specified the follow-up duration by either period ranged from 7 days to 4 months or an event such as intensive care unit admission, hospital discharge, or death. The summary and the baseline features of included RCTs are shown in Tables 1 and 2.

**Table 1:** Summary of the included studies

Study ID	NCT	Vitamin D administration	Follow-up	Primary outcomes	Results
Cannata-Andía et al. (2022)	NCT04552951	Single oral bolus of 100,000 IU of cholecalciferol	Patients were followed up from hospital admission to discharge or death, but not beyond discharge	Biochemical and imaging parameters at discharge	COVID-19 patients who had a cholecalciferol injection at the start of their hospitalizations did not benefit from the treatment
EntrenasCastillo et al. (2020)	NCT04366908	Oral calcifediol in soft capsules (0.532 mg) on days 3 and 7, and then weekly until discharge or ICU admission	Patients were followed up until they were admitted to the ICU, discharged from the hospital, or died	Requirements for admission to the intensive care unit	This research found that large doses of calcifediol dramatically decreased the requirement for ICU therapy in patients with established COVID-19
Fernandes et al. (2022)	NCT04449718	A single oral dose of 200,000 IU of vitamin D3 diluted in vehicle (10 mL of a peanut oil solution)	Ranged from 2 to 4 months	Hospital length stay	The study showed that a single 200,000-IU dosage of vitamin D3 was ineffective in improving cytokines, chemokines, and growth factor levels in individuals with moderate to severe COVID-19 compared with a placebo
Karonova et al. (2022)	NCT05166005	A bolus of cholecalciferol at a dose of 50,000 IU on the 1st and the 8th day of hospitalization, with the total dose being 100,000 IU	Until the ninth day of hospitalization	Changes in serum 25(OH)D level, complete blood count, CRP level in peripheral blood, and B cell subsets	Thus, in vitamin D-deficient and inadequate individuals, an increase in blood 25(OH)D levels mediated by vitamin D supplementation may improve immunological function and thus the course of COVID-19
Maghbooli et al. (2021)	NCT04386850	A dose of 25 mg 25(OH)D3 was administered orally once daily	2 months	Clinical and biochemical outcomes	Vitamin D deficiency/insufficiency in COVID-19 patients was corrected by oral 25(OH)D3 supplementation, which enhanced immunological function by raising blood lymphocyte percentage
Mariani et al. (2022)	NCT04411446	A single oral dose of 500,000 IU of vitamin D3 soft gel capsules (5 capsules of 100,000 IU)	7 days	Change respiratory Sepsis-related Organ Failure Assessment	A single high oral dosage of vitamin D3 could not prevent respiratory deterioration in hospitalized patients with mild-to-moderate COVID-19 and risk factors
Murai et al. (2021a, b)	NCT04449718	A single oral dose of 200,000 IU of vitamin D3 diluted in vehicle (10 mL of a peanut oil solution)	Ranged from 2 to 4 months	Hospital discharge	Compared to a placebo, a single high dosage of vitamin D3 did not substantially shorten the time of stay in the hospital among patients with COVID-19
Rastogi et al. (2022)	NCT04459247	They received 5 mL of cholecalciferol oral solution for 7 days in nanodroplet form	7 days	Change in inflammatory markers	When supplemented with large doses of cholecalciferol, a higher percentage of vitamin D-deficient SARS-CoV-2 patients had negative SARS-CoV-2 RNA and decreased fibrinogen
Soliman et al. (2021)	NCT04733625	Single intramuscular injection of 200,000 units of cholecalciferol	6 weeks	Mortality rate	Taking vitamin D supplements for 6 weeks did not decrease the severity or mortality of COVID-19

*NR* not reported

**Table 2:** Baseline characteristics of the study population

Study ID	Study arms	Sample	Age, years	Sex, males	BMI (kg/m^2^)	Comorbidities					Common COVID symptoms			
						Hypertension	Diabetes	Cardiovascular disease	Asthma	COPD	Fever	Cough	Weakness	Diarrhea
Cannata-Andía et al. (2022)	Cholecalciferol	274	59.3 ± 15.65	181 (66.1)	28.3 ± 3.88	114 (41.6)	58 (21.2)	55 (20.1)	14 (5.1)	14 (5.1)	190 (69.3)	185 (67.5)	167 (60.9)	45 (16.4)
	Control	269	56.3 ± 16.4	172 (63.9)	29 ± 4.85	124 (46.1)	76 (28.3)	60 (22.3)	16 (5.9)	9 (3.3)	198 (73.6)	176 (65.4)	171 (63.6)	60 (22.3)
Entrenas Castillo et al. (2020)	Calcifediol	50	53.14 ± 10.77	27 (54)	NR	11 (24)	3 (6)	2 (4)	NR	4 (8)	NR	NR	NR	NR
	Control	26	52.77 ± 9.35	18 (69)	NR	15 (58)	5 (19)	1 (3.85)	NR	2 (7.69)	NR	NR	NR	NR
Karonova et al. (2022)	Cholecalciferol	56	57.7 ± 11.4	NR	NR	NR	NR	NR	NR	NR	NR	NR	NR	
	Control	54	63 ± 11.4	NR	NR	NR	NR	NR	NR	NR	NR	NR	NR	
Fernandes et al. (2022)	Vitamin D3	101	55.3 ± 14.2	58 (57.4)	32.2 ± 6.7	54 (53.5)	39 (38.6)	14 (13.9)	6 (5.9)	5 (5.0)	73 (72.3)	87 (86.1)	81 (80.2)	33 (32.7)
	Control	99	55.7 ± 14.5	51 (51.5)	32.1 ± 7.5	49 (49.5)	29 (29.3)	13 (13.1)	7 (7.1)	5 (5.1)	69 (69.7)	82 (82.8)	86 (86.9)	40 (40.4)
Maghbooli et al. (2021)	25(OH)D3	53	50 ± 15	32 (59)	29 ± 6	NR	NR	NR	NR	NR	NR	NR	NR	NR
	Control	53	49 ± 13	43 (62)	29 ± 5.5	NR	NR	NR	NR	NR	NR	NR	NR	NR
Mariani et al. (2022)	Vitamin D3	115	59.8 ± 10.7	64 (55.6)	29 ± 5.25	47 (40.9)	32 (27.8)	6 (5.2)	17 (14.8)		80 (69.6)	NR	NR	29 (25.2)
	Control	103	58.3 ± 10.6	51 (49.5)	28.3 ± 4.5	47 (45.6)	26 (25.2)	4 (3.9)	9 (8.7)		68 (66.0)	NR	NR	23 (22.3)
Murai et al. (2021a, b)	Vitamin D3	119	56.5 ± 13.8	70 (58.8)	31.9 ± 6.5	67 (56.3)	49 (41.2)	16 (13.4)	7 (5.9)	7 (5.9)	85 (71.4)	102 (85.7)	97 (81.5)	41 (34.5)
	Control	118	56 ± 15	63 (53.4)	31.4 ± 7.6	58 (49.2)	35 (29.7)	16 (13.6)	7 (5.9)	5 (4.2)	79 (66.9)	97 (82.2)	99 (83.9)	46 (39.0)
Rastogi et al. (2022)	Cholecalciferol	16	45.7 ± 12.19	6 (38)	NR	NR	NR	NR	NR	NR	NR	NR	NR	NR
	Control	24	45.3 ± 7.8	14 (58)	NR	NR	NR	NR	NR	NR	NR	NR	NR	NR
Soliman et al. (2021)	Cholecalciferol	40	71.3 ± 4.16	NR	29.29 ± 2.67	18 (45)	40 (100)	9 (23)	NR	15 (40)	NR	NR	NR	NR
	Control	16	70.19 ± 4.57	NR	29.83 ± 2.19	7 (44)	16 (100)	4 (25)	NR	8 (16)	NR	NR	NR	NR

Data were presented as mean ± standard deviation or number (percentage)

*BMI* body mass index, *NR* not reported

### Risk of bias

**Fig. 2:**
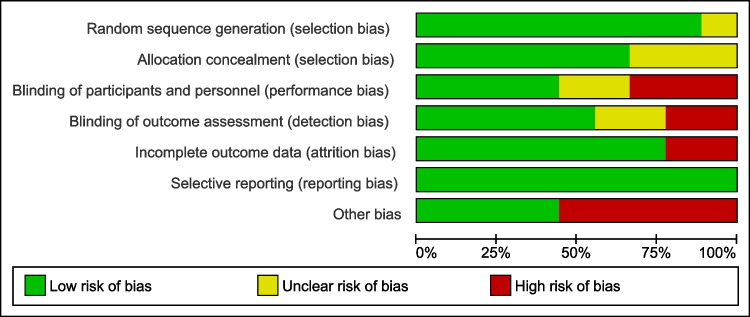
The risk of bias graph of the included studies

The quality of the selected RCTs ranged from moderate to high quality. Eight and six studies were low-biased in random sequence generation and allocation concealment domains, respectively. Participants’ blinding occurred in four trials, while the blinding of outcome assessors occurred in five. Seven studies contained no attrition bias. Reporting bias domain was low-biased in all the included trials. Five studies were judged as high biased regarding other sources of bias domain. The risk of bias graph is presented in Fig. 2.

### Analysis of the outcomes

#### Patients who required ICU admission

**Fig. 3:**
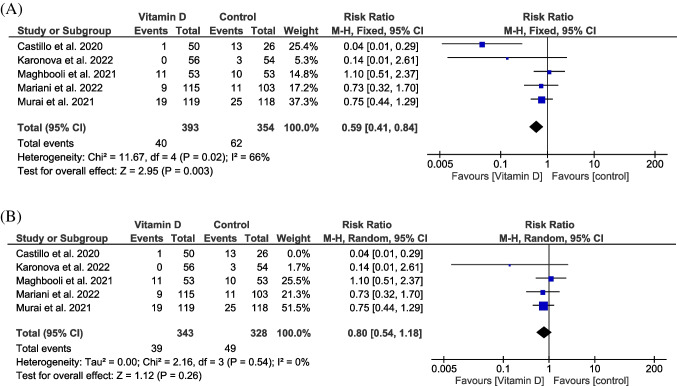
A forest plot for the patients who required ICU admission. **A** Before sensitivity analysis. **B** After sensitivity analysis

Five trials reported this outcome in 671 patients (Entrenas Castillo et al. 2020; Maghbooli et al. 2021; Murai et al. 2021b; Mariani et al. 2022; Karonova et al. 2022). The pooled data showed a significant lower incidence of patients who required ICU admission in vitamin D group compared with placebo group (RR = 0.59, 95% CI [0.41, 0.84], *P* = 0.003) and the pooled analysis was heterogeneous (*P* = 0.02, *I*^2^ = 66%) (Fig. 3A). We used random-effect model and sensitivity analysis by excluding Entrenas Castillo et al. (2020) trial to solve the heterogeneity (*P* = 0.54, *I*^2^ = 0%), and results become insignificant (RR = 0.8, 95% CI [0.54, 1.18], *P* = 0.26) (Fig. 3B).

#### Patients who required ventilation

**Fig. 4:**
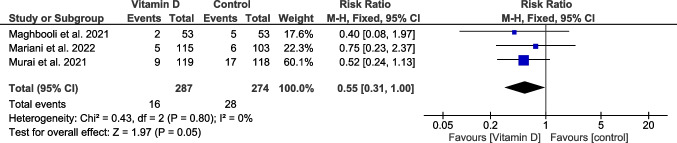
A forest plot for the patients who required ventilation

Totally, 561 patients from three trials reported this outcome (Maghbooli et al. 2021; Murai et al. 2021b; Mariani et al. 2022). The overall estimate showed insignificant superiority of the vitamin D group over the placebo group (RR = 0.55, 95% CI [0.31, 1], *P* = 0.04), and the homogeneity was obvious among trials (*P* = 0.8, *I*^2^ = 0%) (Fig. 4).

#### Patients who required oxygen therapy

**Fig. 5:**
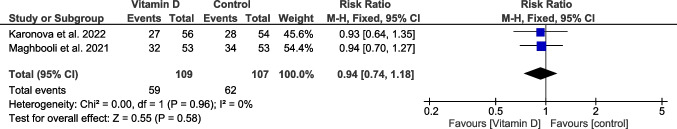
A forest plot for the patients who required oxygen therapy

The pooled data of two studies with 216 participants (Maghbooli et al. 2021; Karonova et al. 2022) revealed insignificant variation between the comparison groups (RR = 0.94 95% CI [0.74, 1.18], *P* = 0.58). The pooled studies were homogeneous (*P* = 0.96, *I*^2^ = 0%) (Fig. 5).

#### Length of hospital stay (days)

**Fig. 6:**
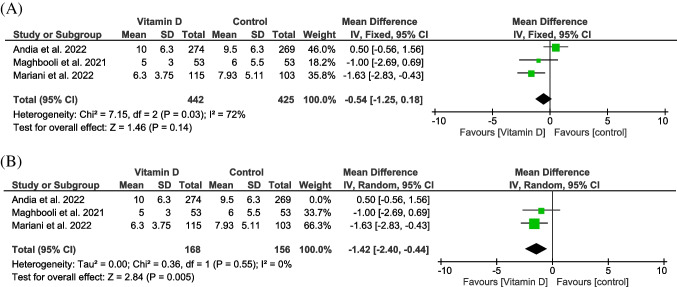
A forest plot for the length of hospital stay (days). **A** Before sensitivity analysis. **B** After sensitivity analysis

Three trials reported this outcome in 867 patients (Cannata-Andía et al. 2022; Maghbooli et al. 2021; Murai et al. 2021b). The intervention group showed insignificant superiority over the control group (MD =  − 0.54, 95% CI [− 1.25, 0.18], *P* = 0.14), and the heterogeneity was detected (*P* = 0.03, *I*^2^ = 72%) (Fig. 6A). Heterogeneity was solved after excluding Cannata-Andía et al. (2022) trial (*P* = 0.55, *I*^2^ = 35%), and results become significant (MD =  − 1.42, 95% CI [− 2.4, − 0.44], *P* = 0.005) (Fig. 6B).

#### Death

**Fig. 7:**
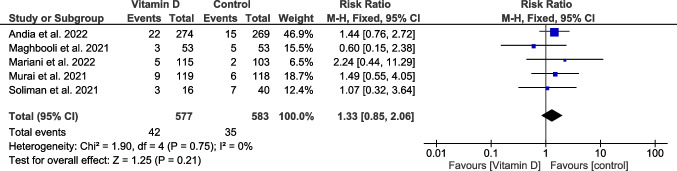
A forest plot for the death incidence

Death was reported by five trials involving 1160 patients (Cannata-Andía et al. 2022; Maghbooli et al. 2021; Murai et al. 2021b; Mariani et al. 2022; Soliman et al. 2021). The overall estimate was non-significant (RR = 1.33, 95% CI [0.85, 2.06], *P* = 0.21), and the homogeneity between trials was observed (*P* = 0.75, *I*^2^ = 0%) (Fig. 7).

#### Change in interleukin-6 (pg/mL)

Three trials with 424 patients reported this outcome (Cannata-Andía et al. 2022; Fernandes et al. 2022; Karonova et al. 2022) and showed a non-significant overall effect size between groups (MD =  − 1.54, 95% CI [− 7.74, 4.67], *P* = 0.63). The analysis was homogeneous (*P* = 0.57, *I*^2^ = 0%) (Suppl. Figure 1).

#### Change in C-reactive protein

Four trials reported this outcome in 690 participants (Cannata-Andía et al. 2022; Fernandes et al. 2022; Rastogi et al. 2022; Karonova et al. 2022), and the intervention group did not show significant results compared to the control group (SMD =  − 0.08, 95% CI [− 0.23, 0.07], *P* = 0.29), and the results were homogeneous (*P* = 0.44, *I*^2^ = 0%) (Suppl. Figure 2).

#### Change in vitamin D

Four trials with 744 patients reported this outcome (Cannata-Andía et al. 2022; Fernandes et al. 2022; Rastogi et al. 2022; Karonova et al. 2022). The vitamin D group showed significant superiority over the placebo group (SMD = 2.27, 95% CI [2.08, 2.47], *P* < 0.00001), and the analysis was heterogeneous (*P* > 0.00001, *I*^2^ = 98%) (Suppl. Figure 3a). Under random-effect model the results were still significant (SMD = 2.62, 95% CI [0.95, 4.29], *P* = 0.002) and the heterogeneity could not be solved by sensitivity analysis (Suppl. Figure 3b).

#### Change in LDH (U/L)

This outcome was reported by two trials in 217 patients (Cannata-Andía et al. 2022; Maghbooli et al. 2021). The effect size showed insignificant change between the vitamin D and placebo groups (MD = 9.93, 95% CI [− 45.57, 65.44], *P* = 0.73), and the analysis was homogeneous (*P* = 0.63, *I*^2^ = 0%) (Suppl. Figure 4).

#### Change in serum calcium (mg/dL)

Three trials with 538 patients reported this outcome (Cannata-Andía et al. 2022; Maghbooli et al. 2021; Murai et al. 2021b). The mean difference revealed no significant results (MD = 0.02, 95% CI [− 0.1, 0.15], *P* = 0.72), and the trials were homogeneous (*P* = 0.36, *I*^2^ = 3%) (Suppl. Figure 5).

#### Change in serum creatinine level (mg/dL)

The effect estimate of three trials with 577 patients (Cannata-Andía et al. 2022; Maghbooli et al. 2021; Murai et al. 2021b) was not significant (MD = 0.02, 95% CI [− 0.04, 0.09], *P* = 0.44), and the data were heterogeneous (*P* = 0.08, *I*^2^ = 60%) (Suppl. Figure 6a). Heterogeneity best solved after Cannata-Andía et al. (2022) trial exclusion (*P* = 0.37, *I*^2^ = 0%), and results stayed insignificant (MD =  − 0.06, 95% CI [− 0.17, 0.04], *P* = 0.23) (Suppl. Figure 6b).

#### Change in d-dimer

Two trials with 277 participants (Murai et al. 2021b; Rastogi et al. 2022) reported insignificant variation between the groups (SMD =  − 0.11, 95% CI [− 0.34, 0.13], *P* = 0.37), and the pooled trials were homogeneous (*P* = 0.69, *I*^2^ = 0%) (Suppl. Figure 7).

#### Change in neutrophil count (× 10^3^/mm^3^)

The overall results of three trials which included 445 participants (Maghbooli et al. 2021; Murai et al. 2021b; Karonova et al. 2022) showed insignificant results (MD =  − 0.29, 95% CI [− 0.65, − 0.07], *P* = 0.11), and the heterogeneity between trials was observed (*P* = 0.07, *I*^2^ = 63%) (Suppl. Figure 8a). We used random-effect model and sensitivity analysis by excluding Karonova et al. (2022) trial to solve the heterogeneity (*P* = 0.3, *I*^2^ = 8%), and results were still insignificant (MD =  − 0.35, 95% CI [− 0.83, 0.12], *P* = 0.14) (Suppl. Figure 8b).

#### Change in lymphocyte count (× 10^3^/mm^3^)

Three trials with 445 patients (Maghbooli et al. 2021; Murai et al. 2021b; Karonova et al. 2022) showed insignificant variation between the study groups (MD =  − 0.04, 95% CI [− 0.28, 0.2], *P* = 0.74), and the analysis was heterogeneous (*P* = 0.03, *I*^2^ = 73%) (Suppl. Figure 9a). We used random-effect model and sensitivity analysis by excluding Maghbooli et al. (2021) trial to solve the heterogeneity (*P* = 0.14, *I*^2^ = 55%), and results were still insignificant (MD =  − 0.03, 95% CI [− 0.39, 0.33], *P* = 0.86) (Suppl. Figure 9b).

#### Change in platelet count (× 10^3^/mm^3^)

The estimate of two trials with 340 patients (Maghbooli et al. 2021; Murai et al. 2021b) showed no significant favor of the intervention over the control group (MD =  − 5.63, 95% CI [− 41.39, 30.12], *P* = 0.76), and the pooled analysis was homogeneous (*P* = 0.83, *I*^2^ = 0%) (Suppl. Figure 10).

#### Change in leucocytes (no./μL)

Three studies with 657 participants (Cannata-Andía et al. 2022; Fernandes et al. 2022; Maghbooli et al. 2021) reported this outcome and the results were non-significant (MD =  − 0.19, 95% CI [− 0.8, 0.42], *P* = 0.55) (Suppl. Figure 11a). Heterogeneity between the groups was observed (*P* = 0.06, *I*^2^ = 64%), and solved after excluding Maghbooli et al. (2021) (*P* = 0.73, *I*^2^ = 0%). Under random-effect model, the results were still insignificant (MD = 0.16, 95% CI [− 0.52, 0.83], *P* = 0.65) (Suppl. Figure 11b).

## Discussion

This systematic review and meta-analysis of nine RCTs aimed to find a definitive role of vitamin D on hospital and laboratory outcomes of COVID-19 patients. The analysis showed a significantly reduced risk of ICU admission. Also, vitamin D3 levels significantly affect its level positively. However, administration of vitamin D showed no significant difference compared to placebo regarding most hospital-related outcomes of the COVID-19 disease, including requiring ventilation, requiring oxygen therapy, death rate, and length of hospital stay. As for laboratory outcomes, a non-significant difference was also detected in the change in levels of interleukin-6, C-reactive protein, LDH, serum calcium, serum creatinine, d-dimer, neutrophil count, lymphocyte count, platelet count, and leucocytic count.

As for the ICU admission, our results showed a significant reduction in COVID-19 patients who received vitamin D. However, after solving the heterogeneity, the results turned non-significant. Our results were supported by another meta-analysis that concluded the positive effect of vitamin D on ICU admission; however, this study included observational studies, which may affect the results (Shah et al. 2021). Another meta-analysis of six studies suggested the influential role of vitamin D in ICU admission (Tentolouris et al. 2022). In another RCT, a significantly lower likelihood of ICU admission was maintained even after correcting for comorbidities such as hypertension and diabetes (Entrenas Castillo et al. 2020).

The previously mentioned results differ from Rawat et al., which excluded the retrospective study and found a non-significant effect on ICU admission (Rawat et al. 2021). The first multicenter, double-blind RCT in moderate-severe COVID-19 patients concluded that receiving a single high dosage of vitamin D3 (200,000 IU orally) did not lower the ICU admission, length of hospital stay, or rates of mechanical ventilation compared to peanut oil (Murai et al. 2021b). In another multicenter RCT on mild-moderate COVID-19 patients, insignificant changes in ICU or mortality events were observed even though the vitamin D arm had a considerably quicker recovery time to symptoms (even after controlling for age, gender, BMI, and d-dimer) (Sabico et al. 2021). The variations between the abovementioned studies may be due to the different comorbidities, the standard of care, severity of COVID-19, and vitamin D levels at the beginning of each trial. Regarding ventilation, previous studies reported inconsistent results with ours (Rawat et al. 2021; Bassatne et al. 2021; Murai et al. 2021a, b; Maghbooli et al. 2021). However, Maghbooli et al. concluded that vitamin D would benefit COVID-19 patients despite the insignificant results (Maghbooli et al. 2021).

Previous studies supported our results regarding death from COVID-19 and found that vitamin D did not reduce mortality (Tentolouris et al. 2021; Bassatne et al. 2021; Shah et al. 2021; Rawat et al. 2021; Cannata-Andía et al. 2022; Hernández et al. 2021; Murai et al. 2021b; Sabico et al. 2021; Beran et al. 2022). In contrast, Varikasuvu et al. reported that vitamin D significantly reduces mortality (Varikasuvu et al. 2022a). Other studies also reported a significant reduction in mortality favoring vitamin D over placebo (Nikniaz et al. 2021; Pal et al. 2022). Furthermore, a positive association between vitamin D insufficiency and the increased mortality from COVID-19 was detected, especially in the elderly (Pereira et al. 2022). This was explained by lower exposure to the sun, lower levels of 7-dehydrocholesterol in the skin, higher risk of severe COVID-19 due to comorbidities, and interference of vitamin D levels by the drugs used to treat these comorbidities (Adami et al. 2009; Pimenta et al. 2015; Grant et al. 2020; Jin et al. 2020). Also, Drame et al., in their systematic review, suggested an association between vitamin D deficiency and increased risk of COVID-19 positivity, unfavorable disease course, bad outcomes regarding mortality, disease severity, oxygen therapy requirements, and ventilation need (Dramé et al. 2021).

Elamir et al. reported that the intervention group did not affect the length of hospital stay and intubation need, which supports our results; however, they reported a significant reduction in oxygen therapy requirements, which is inconsistent with ours (Elamir et al. 2022). They explained this by the small number of participants in the trial but suggested a beneficial role of vitamin D on respiration (Elamir et al. 2022). A recent meta-analysis reported that vitamin D benefits both length of hospital stay and intubation requirements, which contrasts with our results (Beran et al. 2022). Another cohort analysis of the length of hospital stay and the death rate showed superiority in the highest serum calcidiol group (> 25 ng/mL) (Cannata-Andía et al. 2022). However, Bassatne et al. reported insignificant results (Bassatne et al. 2021). So, determination of any vitamin D deficiency in any patients is mandatory as the baseline vitamin D level would influence the benefits of its supplementation and the COVID-19 outcomes (Griffin et al. 2020).

Hypercalcemia was not observed in our included studies either in intervention or control groups, which means no difference between groups and proves the safety of vitamin D on the calcium level (Elamir et al. 2022; Rastogi et al. 2022). Previous research found a significant increase from baseline in vitamin D levels after vitamin D3 supplementation, consistent with our results (Fernandes et al. 2022; Rastogi et al. 2022; Murai et al. 2021b; Soliman et al. 2021). In similarity to our results, other studies reported insignificant results regarding d-dimer, CRP, IL-6, and LDH levels (Rawat et al. 2021; Rastogi et al. 2022; Fernandes et al. 2022; Maghbooli et al. 2021). It is known that COVID-19 raises inflammatory markers like d-dimer, fibrinogen, IL-6, and CRP, especially in severe cases, which are considered good indicators for severity and recovery of COVID-19 (Velavan and Meyer 2020).

Previous studies reported consistent results with ours regarding serum creatinine levels (Cannata-Andía et al. 2022; Maghbooli et al. 2021; Murai et al. 2021b). Furthermore, similar to our results regarding the change in the count of platelets, lymphocytes, and leucocytes, some researchers reported insignificant results, but others reported significant results regarding lymphocytic count (Maghbooli et al. 2021; Murai et al. 2021b). In response to inflammation such as COVID-19 events, leukocytes provide innate immunity, and lymphocytes provide adaptive immunity, so body defense occurs (Denman 1979).

Research proved that vitamin D exerts a biological effect in modulating the innate immune response, regulating the adaptive immune response, interacting with the renin‐angiotensin‐aldosterone system, protecting the endothelial functions, and yielding an antithrombotic action (Charoenngam et al. 2021; Griffin et al. 2020; Bilezikian et al. 2020; Arnold 2020; Malek Mahdavi 2020). These mechanisms reduce cytokine storm risk, enhance the immune response, and produce antiinflammatory, antiviral, and antimicrobial activities (Mercola et al. 2020; Teymoori-Rad et al. 2019; Pinheiro et al. 2021; Malek Mahdavi 2020; Gois et al. 2017). These protective functions of vitamin D were observed in patients with respiratory diseases (Jolliffe et al. 2021; Lips 2021; AlSafar et al. 2021) and patients who received the COVID-19 vaccine (Velikova et al. 2021; Chiu et al. 2021).

An acute illness such as COVID-19 reduces the circulation of vitamin D binding protein and interferes with the effective production of the body’s active form of vitamin D (Zehnder et al. 2001; Waldron et al. 2013). These phenomena may help explain the conflict between studies regarding the effectiveness of vitamin D on COVID-19.

Our study has several strengths which support the quality of the evidence. For example, we applied a comprehensive search strategy and literature search on different databases without language or time restrictions. We included only relevant RCTs that studied clinical and laboratory outcomes and excluded any other design. The included trials are considered low-biased regarding many quality assessment domains, which is supportive.

However, we found high heterogeneity between the included studies, such as different populations’ characteristics, including age, sex, race, body mass index, general status, the severity of COVID-19 symptoms, treatment protocol of the patients, and associated comorbidities. The regimens of vitamin D supplementation also varied across the studies regarding the form, the dose, the timing of administration, and the baseline levels of vitamin D. Patients received variable amounts of vitamin D, ranging from low to high doses and from single to daily doses. Previous research found that the daily doses of vitamin D prevent and treat certain diseases such as acute respiratory infections, rickets, and tuberculosis better than the intermittent doses (Griffin et al. 2021). Most of the studies included a low sample size, which also affected the quality of the evidence. During acute illness, vitamin D binding protein and albumin tend to decrease by the negative acute phase response, which affects vitamin D levels bound to them (Rhodes et al. 2021). Time of vitamin D administration also would impact its effect as most patients have received it after being infected and diagnosed with COVID-19.

## Conclusions

Our study suggested that vitamin D supplementation benefits COVID‐19 patients by reducing ICU admission and increasing changes in vitamin D levels. However, it produces no difference in other outcomes compared to no vitamin D intake. The definite role of vitamin D on COVID‐19 outcomes strongly needs further well-conducted and high-quality research, especially after its known effect on the body’s immune system and defense mechanisms and the previously collected data on its benefits on certain respiratory diseases, including COVID-19.

## Supplementary Information

Below is the link to the electronic supplementary material.

Supplementary file1 (DOCX 159 KB)

## Data Availability

The datasets generated during and/or analyzed during the current study are available from the corresponding author on reasonable request.
