# Supplementary material for: Hospital and laboratory outcomes of patients with COVID-19 who received vitamin D supplementation: a systematic review and meta-analysis of randomized controlled trials
Source: Naunyn Schmiedebergs Arch Pharmacol. 2022 Dec 12;396(4):607–20. doi: 10.1007/s00210-022-02360-x (PMC9743115; doi:10.1007/s00210-022-02360-x)

Suppl. Fig. 1. A forest plot for the change in interleukin-6 (pg/mL).


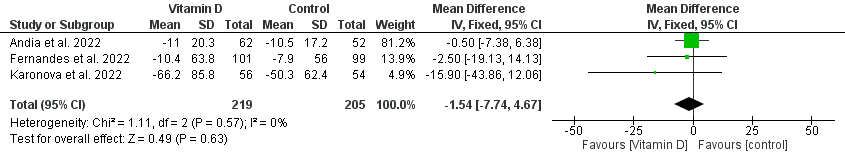


Suppl. Fig. 2. A forest plot for the change in C-reactive protein.


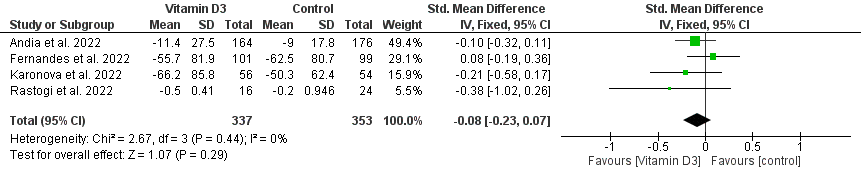


Suppl. Fig. 3. A forest plot for the change in vitamin D.

(A) Before sensitivity analysis


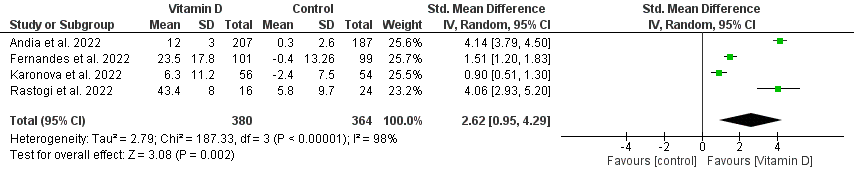


(B) After sensitivity analysis


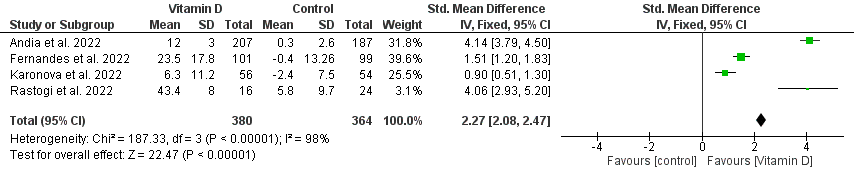


Suppl. Fig. 4. A forest plot for the change in LDH (U/L).


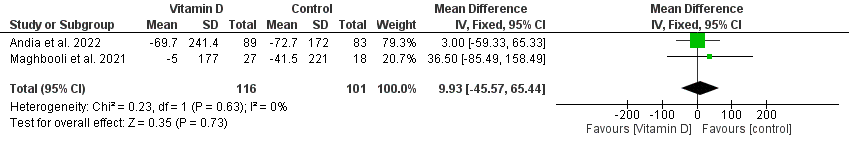


Suppl. Fig. 5. A forest plot for the change in serum calcium (mg/dL).


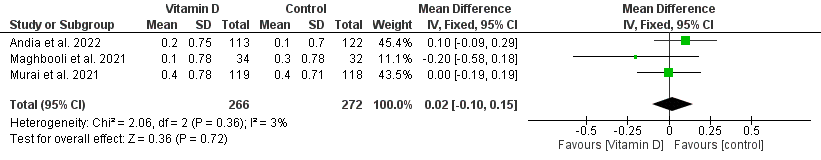


Suppl. Fig. 6. A forest plot for the change in serum creatinine level (mg/dl).

(A) Before sensitivity analysis


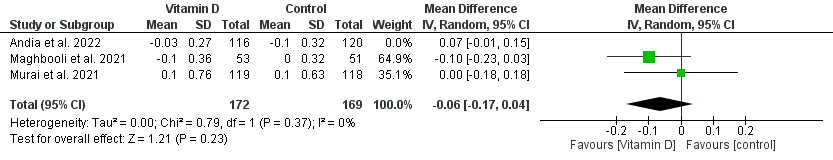


(B) After sensitivity analysis


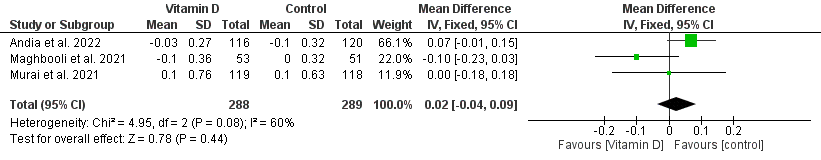


Suppl. Fig. 7. A forest plot for the change in d-dimer.


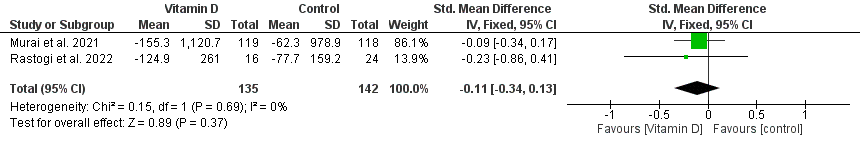


Suppl. Fig. 8. A forest plot for the change in neutrophils count (x10³/mm³).

(A) Before sensitivity analysis


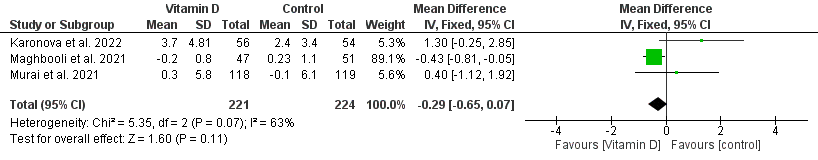


(B) After sensitivity analysis


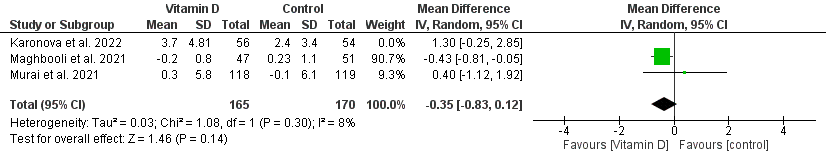


Suppl. Fig. 9. A forest plot for the change in lymphocyte count (x10³/mm³).

(A) Before sensitivity analysis


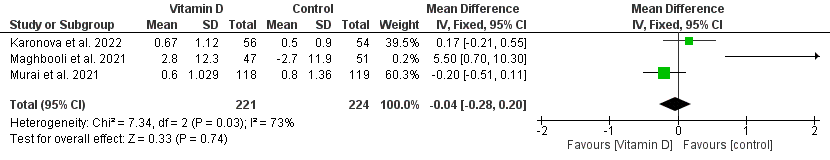


(B) After sensitivity analysis


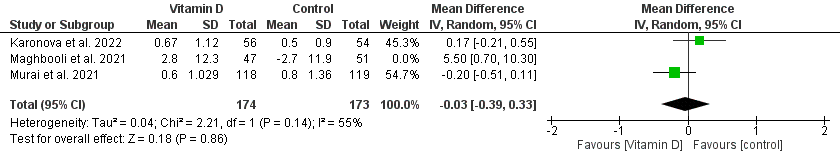


Suppl. Fig. 10. A forest plot for the change in platelet count (x10³/mm³).


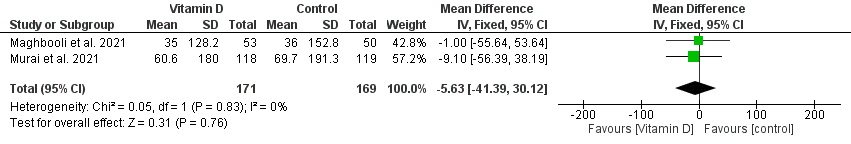


Suppl. Fig. 11. A forest plot for the change in leucocytes (No./μL).

(A) Before sensitivity analysis


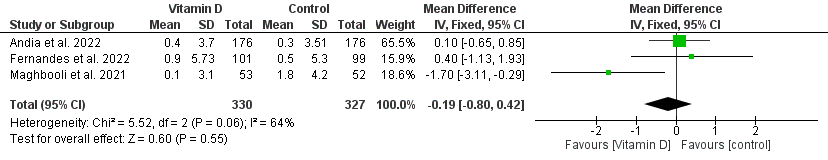


(B) After sensitivity analysis


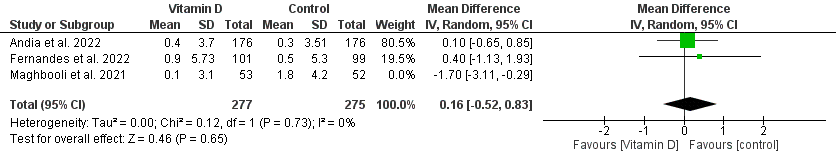

Supplement: Supplementary file 1 — Supplementary file1 (DOCX 159 KB) [file 210_2022_2360_MOESM1_ESM.docx]
